# Supplementary material for: Complex phase transitions and phase engineering in the aqueous solution of an isopolyoxometalate cluster
Source: Nat Commun. 2023 May 13;14:2767. doi: 10.1038/s41467-023-38455-z (PMC10183013; doi:10.1038/s41467-023-38455-z)
Supplement: Supplementary file 1 — Supplementary Information [file 41467_2023_38455_MOESM1_ESM.docx]

**Supplementary Information**

**Complex phase transitions and phase engineering in the aqueous solution of an isopolyoxometalate cluster**

**Zhi-Da Wang^1,2#^, Song Liang^2#^, Yuqing Yang^4^, Zhen-Ning Liu^2^, Xiao-Zheng Duan^3🖂^, Xinpei Li^5^, Tianbo Liu^4🖂^, Hong-Ying Zang^1🖂^**

^1^Key Laboratory of Polyoxometalate and Reticular Science of the Ministry of Education, Faculty of Chemistry, Northeast Normal University, Changchun 130024, China. ^2^Key Laboratory of Bionic Engineering (Ministry of Education), College of Biological and Agricultural Engineering, Jilin University, Changchun 130022, China. ^3^State Key Laboratory of Polymer Physics and Chemistry, Changchun Institute of Applied Chemistry, Chinese Academy of Sciences, Changchun 130022, China. ^4^School of Polymer Science and Polymer Engineering, The University of Akron, Akron, Ohio 44325, United States. ^5^South China Advanced Institute for Soft Matter Science and Technology, Guangdong Provincial Key Laboratory of Functional and Intelligent Hybrid Materials and Devices, South China University of Technology, Guangzhou 510640, China. **^#^**The authors contributed equally. **^🖂^**e-mail: tliu@uakron.edu; xzduan@ciac.ac.cn; zanghy100@nenu.edu.cn

***Contents:***

***Supplementary Figures 1-6***

**Supplementary Figures**


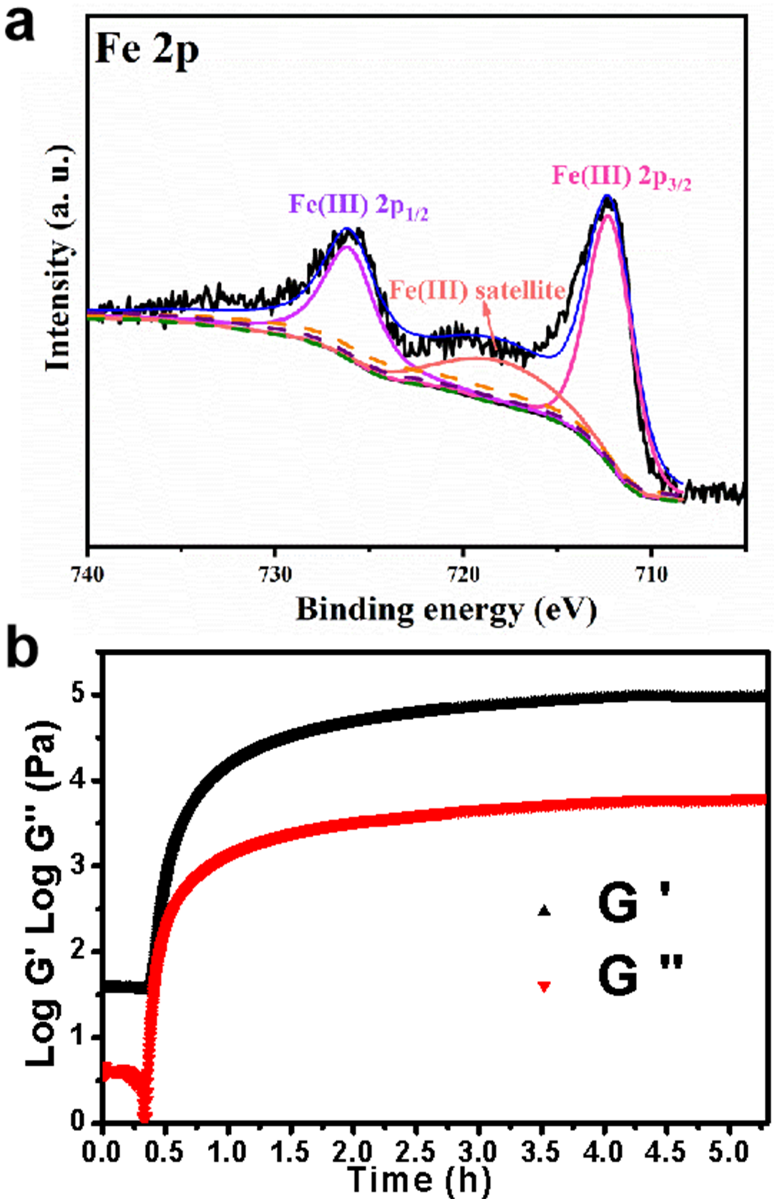


**Supplementary Fig. 1.** (a) The XPS spectra for Fe 2p of Mo7-Fe hyfrogel. (b) Storage modulus (G′) and loss modulus (G″) of the Mo7-Fe system as a function of time.


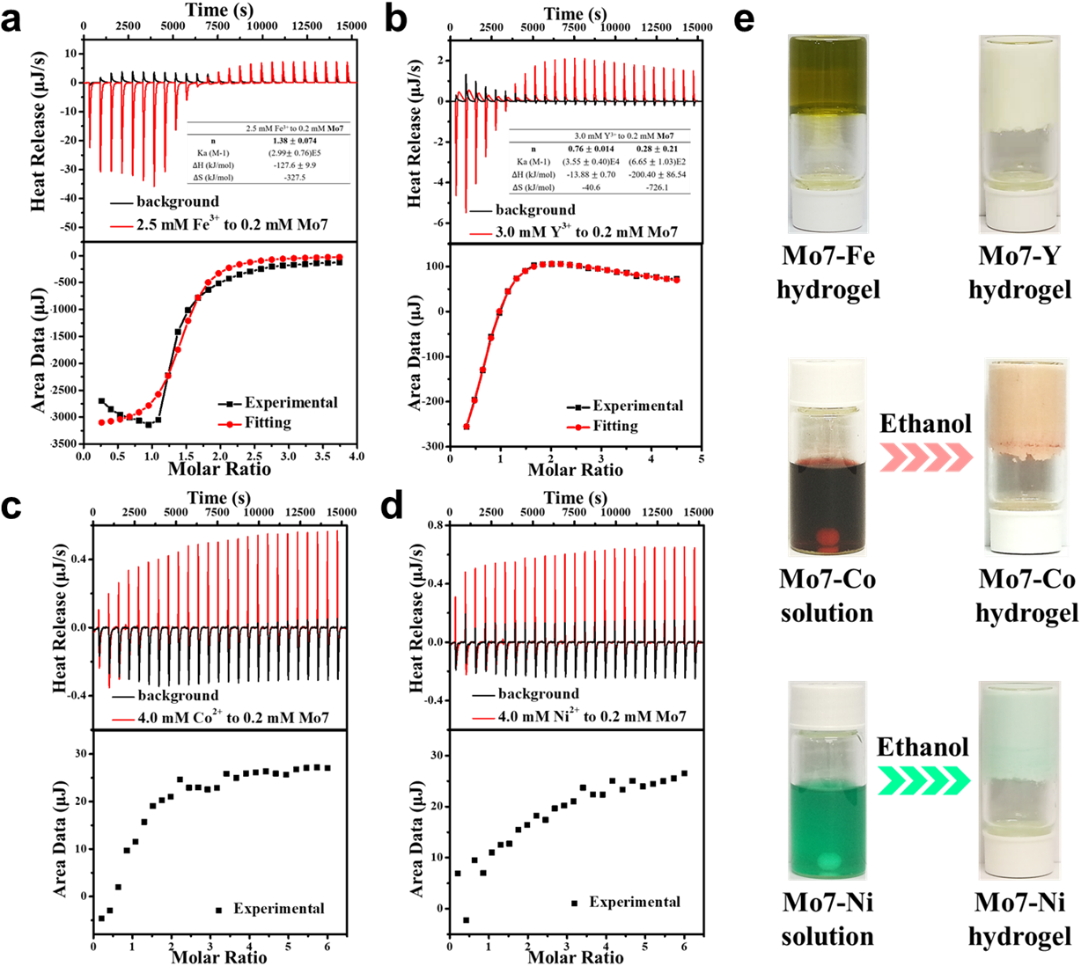


**Supplementary Fig. 2.** ITC results of (a) Mo7-Fe, (b) Mo7-Y, (c) Mo7-Co, and (d) Mo7-Nisystem. (e) The corresponding photograph in these systems.


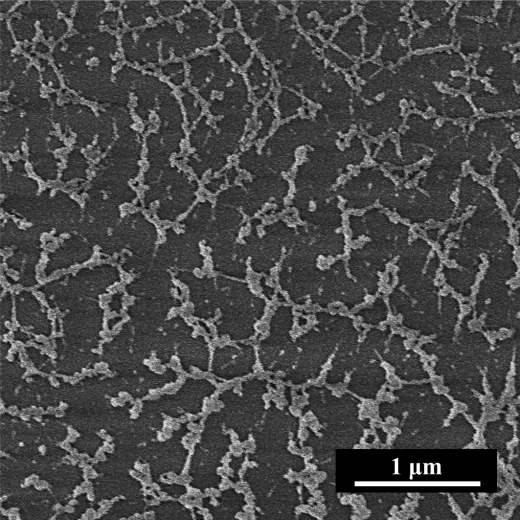


**Supplementary Fig. 3.** SEM of freeze-dried Mo7-Fe gel.


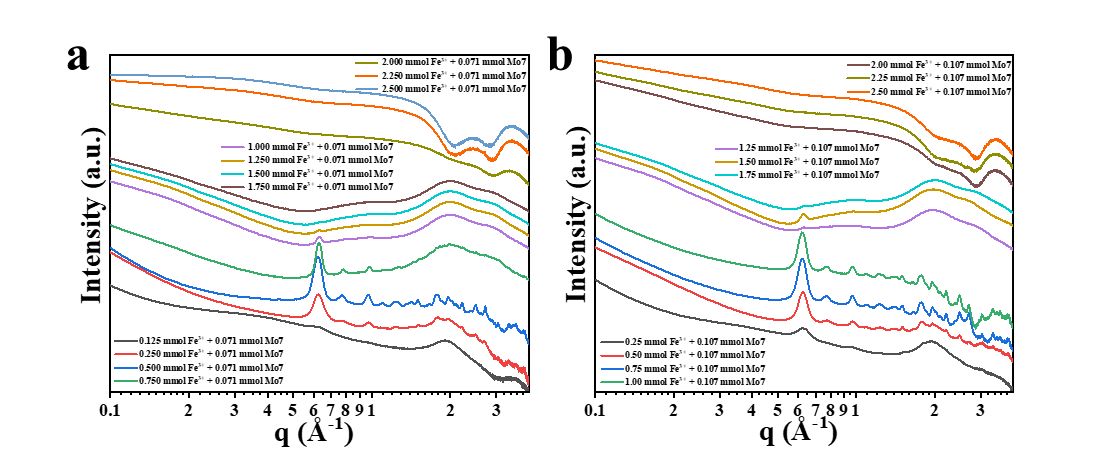


**Supplementary Fig. 4.** SAXS spectra within the Mo7-Fe system.


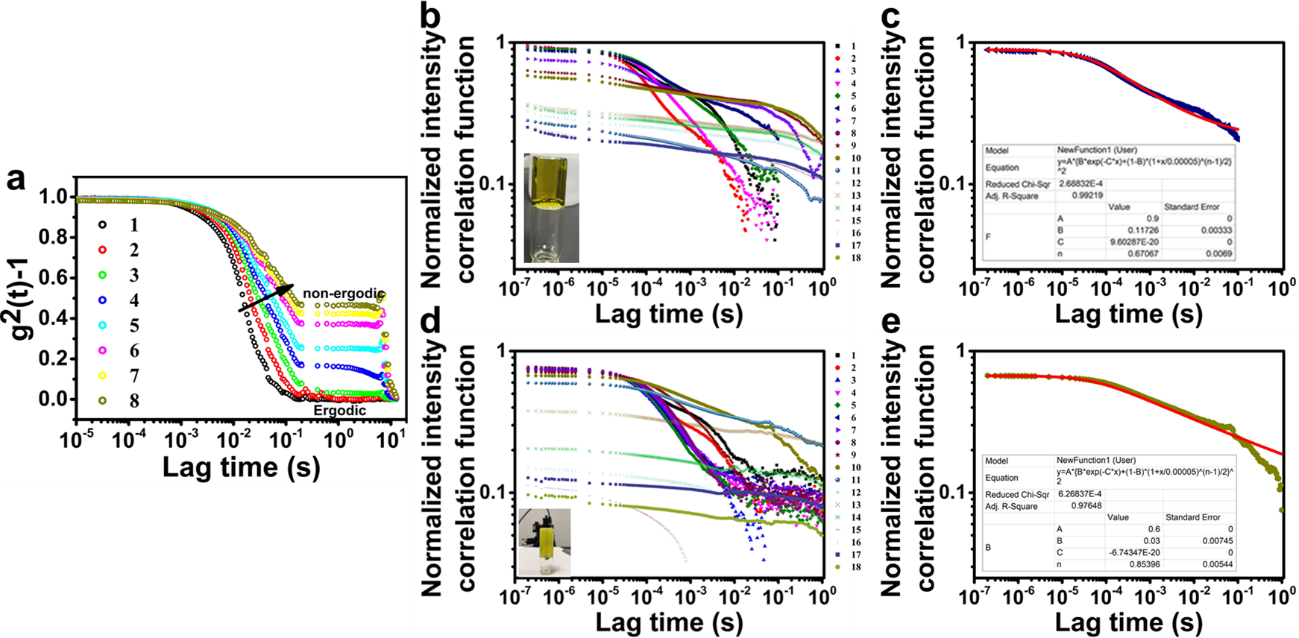


**Supplementary Fig. 5.** (a) The DWS results from the *in situ* backscattering experiments. (b, d) Intensity correlation function and (c, e) a power-low behavior in ICF of transparent hydrogel and turbid hydrogel, respectively.


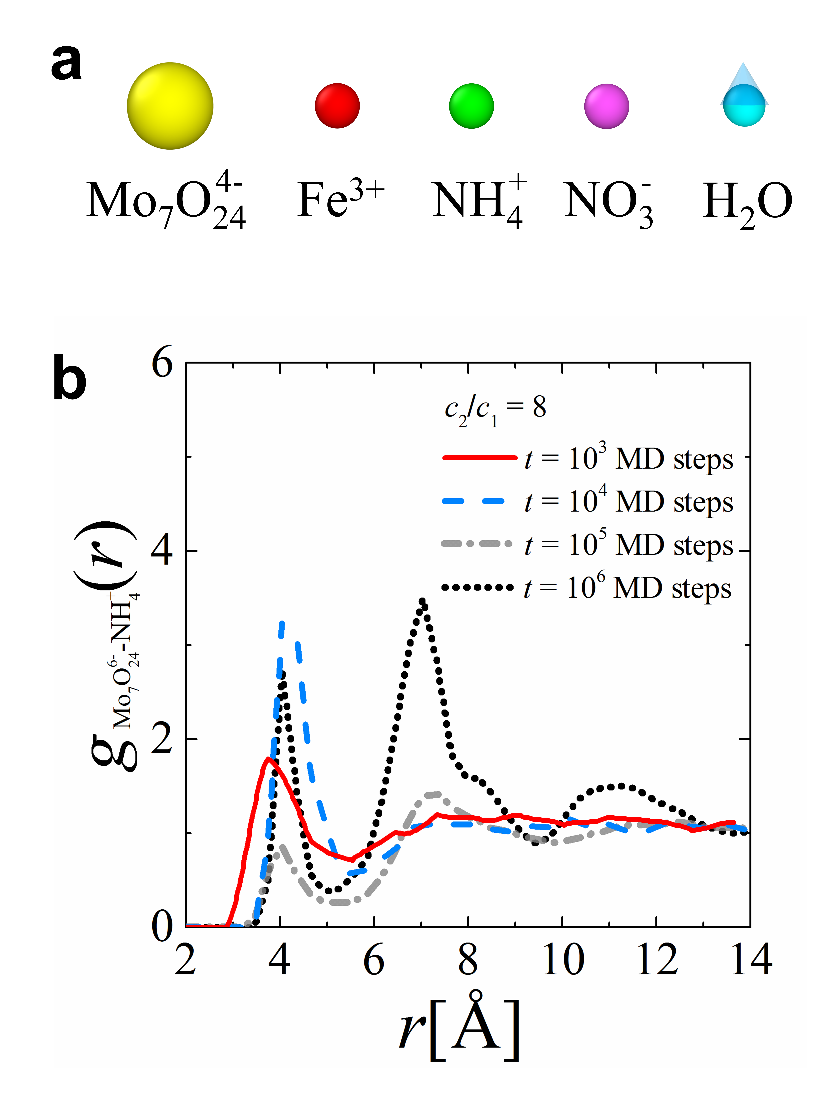


**Supplementary Fig. 6.** (a) Schematics of coarse-grained model for $\mathrm{Mo}_{7}O_{24}^{6-}$, Fe^3+^, $\mathrm{NH}_{4}^{+}$, $\mathrm{NO}_{3}^{-}$ and H_2_O (the blue triangle indicates the dipole vector of H_2_O molecules). (b) Radial distribution functions of **Mo7** around NH+ 4 at different simulation time steps.

**Molecular Dynamics (MD) Simulations**

The coarse-grained simulations in statistical mechanics typically consist of a minimal set of model parameters and they are, therefore, considered a robust strategy that is well suited for exploring ion-containing systems. We employ coarse-grained molecular dynamics (MD) simulations to study the mechanisms for ionic gel formation in the (NH_4_)_6_Mo_7_O_24_/Fe(NO_3_)_3_ mixed solutions. As shown in Supplementary Fig. 6a, we coarse-grain the ionic species and water molecules as spherical beads and draw upon the Stockmayer fluid model to consider the dipolar nature of the water molecules. Given that the electrostatic correlations and ionic solvation serve as the main driving force for the complex phase transition discussed in the current work, our coarse-grained model can effectively capture the coupling of these interactions. By neglecting the detailed molecular structures, our simulations are independent of any atomistic force field and can reflect the structural evolutions in relatively large time and length scales. Therefore, the systematic coarse-grained simulations are suitable for studying the sol-gel transitions of $\mathrm{NH}_{4}\mathrm{Mo}_{7}O_{24}$/$\mathrm{Fe}{(\mathrm{NO}_{3})}_{3}$ mixed solutions and could be used to clarify the underlying mechanism on the molecular level.

We account for the excluded volume interactions between the particles through a truncated and shifted purely repulsive Lennard-Jones (LJ) potential,

$U_{ij}^{LJ}\left( r_{ij} \right)=\left\{ \begin{aligned} 4\varepsilon_{\mathrm{LJ}}\left[ \left( \frac{\sigma}{r_{ij}} \right)^{12}-\left( \frac{\sigma}{r_{ij}} \right)^{6} \right]+\varepsilon_{LJ}, r_{ij}\leq r_{c} \\ 0,r_{ij}>r_{c} \end{aligned} \right.$, Eq. S1

where *ε*_LJ_ and $\sigma$ represent the energy parameters and diameters of the particles, respectively, and $r_{ij}=\left| \vec{r}_{i}-\vec{r}_{j} \right|$ is the separation distance between particles *i* and *j*. Herein, we vary the energy parameters between different species from *ε*_LJ_ =1.0 to 2.25 in LJ units (which corresponds to 1-2 *k*_B_*T* at room temperature). For simplicity, the diameter of **Mo7** is set as *σ* = 2.0 (which corresponds to 6 [Å]) and the diameters of other species are set as *σ* = 1.0 (which corresponds to 3 [Å]) in LJ units. The cutoff is varied from *r_c_* = 1.12246 to 2.5 in LJ units, and the LJ potential is shifted to 0 at *r_c_* by the factor *S*. The electrostatic interactions between ion and ion, ion and dipole, and dipole and dipole can be written as,

$U_{mn}^{ii}\left( r_{mn} \right)=-\frac{q_{m}q_{n}e^{2}}{4\pi\epsilon_{0}r_{mn}}$, Eq. S2

$U_{mj}^{id}\left( r_{mj} \right)=\frac{q_{m}e}{4\pi\epsilon_{0}}\frac{\vec{\mu}_{j}\cdot\vec{r}}{r_{mj}^{3}}$, Eq. S3

$U_{ij}^{dd}\left( r_{ij} \right)=\frac{1}{4\pi\epsilon_{0}r_{ij}^{3}}\left[ \vec{\mu}_{i}\cdot\vec{\mu}_{j}-\frac{3(\vec{\mu}_{i}\cdot\vec{r})(\vec{\mu}_{j}\cdot\vec{r})}{r_{ij}^{2}} \right]$, Eq. S4

Here, *q_m_* and *q_n_* represent the charge of ion *m* and *n*, $\vec{\mu}_{i}$ and $\vec{\mu}_{j}$ denote the dipole moments of solvent particles *i* and *j*, *e* is the elementary charge, and *ε_0_* denotes the vacuum permittivity. The long-range electrostatic interactions described in Equations S2, S3, and S4 are taken into account through Ewald summation.

We perform the simulations in a cubic box of length *L* = 15 (which corresponds to 45 [Å]) with 3D periodic boundary conditions in the canonical (NVT) ensemble using the Large-scale Atomic/Molecular Massively Parallel Simulator (LAMMPS). We use a time step of Δ*t* = 0.002τ (corresponding to 2 fs), where τ= *σ*(*m*/*ε*_LJ_)^0.5^, and set the temperature as *T* = 1.0 controlled by the Nosé–Hoover thermostat, which corresponds to the room temperature 298K. The number density of all species is set to be *ρ* ~ 0.8. We set the dipolar solvent molecules from *μ* = 1.85 [D], which corresponds to the dipole moment of H_2_O molecules. We randomly generate the ions and solvent molecules in the simulation box and perform the order of 10^6^ time steps (corresponding to 2 ns) for production simulations, which has proven to be quite long enough for ionic gel formation. For each case, we perform scores of simulations with different initial configurations and obtain the final results from the order of 10^2^ statistically independent samples. In the simulation, we set the concentration of (NH_4_)_6_Mo_7_O_24_ as *c*_1_ = 0.5 [M] (*i.e.*, 30 **Mo7** particles and 180 NH+ 4 particles in the simulation box), and vary the concentration ratio of Fe(NO_3_)_3_ and (NH_4_)_6_Mo_7_O_24_ from *c*_2_/*c*_1_ from 1 to 1.6, which has been tested effective for the modeling study of ionic gel formation.
